# The Anoikis Effector Bit1 Displays Tumor Suppressive Function in Lung Cancer Cells

**DOI:** 10.1371/journal.pone.0101564

**Published:** 2014-07-08

**Authors:** Xin Yao, Scott Jennings, Shubha Kale Ireland, Tri Pham, Brandi Temple, Mya Davis, Renwei Chen, Ian Davenport, Hector Biliran

**Affiliations:** 1 Center for Nanomedicine, Sanford-Burnham Medical Research Institute, University of California Santa Barbara, Santa Barbara, California, United States of America; 2 Department of Molecular, Cell and Developmental Biology, University of California Santa Barbara, Santa Barbara, California, United States of America; 3 Department of Biology, Xavier University of Louisiana, New Orleans, Louisiana, United States of America; Thomas Jefferson University, United States of America

## Abstract

The mitochondrial Bit1 (Bcl-2 inhibitor of transcription 1) protein is a part of an apoptotic pathway that is uniquely regulated by integrin-mediated attachment. As an anoikis effector, Bit1 is released into the cytoplasm following loss of cell attachment and induces a caspase-independent form of apoptosis. Considering that anoikis resistance is a critical determinant of transformation, we hypothesized that cancer cells may circumvent the Bit1 apoptotic pathway to attain anchorage-independence and tumorigenic potential. Here, we provide the first evidence of the tumor suppressive effect of Bit1 through a mechanism involving anoikis induction in human lung adenocarcinoma derived A549 cells. Restitution of Bit1 in anoikis resistant A549 cells is sufficient to induce detachment induced-apoptosis despite defect in caspase activation and impairs their anchorage-independent growth. Conversely, stable downregulation of Bit1 in these cells significantly enhances their anoikis resistance and anchorage-independent growth. The Bit1 knockdown cells exhibit significantly enhanced tumorigenecity *in vivo*. It has been previously shown that the nuclear TLE1 corepressor is a putative oncogene in lung cancer, and we show here that TLE1 blocks Bit1 mediated anoikis in part by sequestering the pro-apoptotic partner of Bit1, the Amino-terminal Enhancer of Split (AES) protein, in the nucleus. Taken together, these findings suggest a tumor suppressive role of the caspase-independent anoikis effector Bit1 in lung cancer. Consistent with its role as a tumor suppressor, we have found that Bit1 is downregulated in human non-small cell lung cancer (NSCLC) tissues.

## Introduction

A critical determinant of a malignant epithelial phenotype is anchorage independence. The loss of anchorage dependence to the extracellular matrix by malignant cells enables them to grow in the absence of cell adhesion, to propagate in a three dimensional matrix, to invade adjacent tissues, and metastasize to distant organs [1], [2]. The molecular mechanisms and cellular pathways underlying anchorage-independence of cancer cells have been extensively studied [3], , and most of these molecular alterations confer malignant cells the ability to evade the anoikis program which is in place in normal cells. Considering that breakdown of anoikis control contributes to growth and malignancy of many solid tumors, restoring anoikis sensitivity represents an important therapeutic strategy in curtailing tumor aggressiveness and metastasis. Hence, identification of novel molecular targets of the anoikis-mediated cell death pathway has significant therapeutic implications.

Two apoptotic pathways, the mitochondrial (intrinsic) and cell death receptor (extrinsic), have been shown to regulate the anoikis process. Following detachment-induced loss of integrin-mediated survival signalling, the intrinsic apoptotic pathway is triggered through activation of the pro-apoptotic members of the BCL family of proteins (including Bax, Bad, Bid and Bim). These proteins trigger permeabilization of the outer mitochondrial membrane leading to cytosolic release of cytochrome c and downstream activation of caspase enzymes [5], [6]. This mitochondrial-dependent caspase activation loop, which can be further amplified via downregulation of expression of the anti-apoptotic Bcl family members (such as Bcl-2 and Bcl-xL) which function in guarding mitochondrial integrity [7], may ultimately lead to DNA fragmentation and cell death. The death receptor (extrinsic) pathway depends upon the binding of death ligands (Fas or TRAIL) to death receptor and utilizes the formation of a death-inducing signaling complex (DISC) in activating the downstream caspase-8. This death receptor-mediated caspase 8 activation may effect destabilization of the mitochondrial membrane leading to apoptosis [8], [9]. Although convergence and crosstalk between extrinsic and intrinsic apoptotic pathways exist at various levels, both pathways rely on caspase activation loop to effect cell death. However, evidences have emerged indicating the existence of caspase-independent mechanisms in anoikis [10], . In particular, inhibition of caspase activation either through overexpression of Bcl-2 or treatment with global caspase inhibitors is unable to block basal anoikis in tumor cells as assessed by DNA ladder analysis [10]. The alternative caspase-independent mode of anoikis is an important therapeutic target in tumors that exhibit a disabled caspase-dependent apoptosis pathway.

Ruoslahti and colleagues have recently identified a novel integrin-dependent apoptotic pathway that is mediated by the mitochondrial Bit1 (Bcl2, inhibitor of transcription 1) protein [11]. Following loss of integrin-mediated cell attachment, Bit1 is released into the cytoplasm, forms a complex with the transcriptional coregulator AES, and subsequently induces a caspase-independent mode of apoptosis. While several known anti-apoptotic factors such as Bcl-2, Bcl-xl, Akt are ineffective in blocking Bit1 apoptosis, integrin-mediated attachment is the sole anti-apoptotic treatment that can suppress Bit1 apoptosis function [11]. Considering that integrin-mediated cell adhesion, particularly the α5β1 integrin, is the only up-stream treatment that can block the Bit1 apoptosis pathway, Bit1 may play an important role in anoikis as a guardian of anchorage dependence [11]–[15]. Based on the inability of caspase inhibitors to inhibit Bit1 apoptosis and the absence of caspase activation in Bit1 transfected cells, the Bit1 pathway may represent one of the caspase-independent anoikis mechanisms in malignant cells [11]. Hence, the Bit1 apoptotic pathway appears to be an attractive therapeutic target in circumventing anoikis resistance particularly in tumor cells that exhibit deficient caspase activity. Utilization of Bit1 as a therapeutic target requires a detailed examination of the mechanism of its apoptosis function, which we have found to be dependent in part on turning off a survival-promoting gene transcription program controlled by the groucho transcriptional corepressor TLE1 [15].

The role of Bit1 in anoikis has been demonstrated in several transformed cell lines. Through genetic manipulation approaches, we have shown that exogenous expression of mitochondrial Bit1 blocks the anoikis resistance of several tumor cell lines while downregulation of endogenous Bit1 expression further confounds their anoikis insensitivity [11]–[15]. Since the acquisition of anoikis resistance is an important determinant of neoplastic transformation and metastatic potential [1], [2], suppression of the Bit1 anoikis pathway in malignant cells may contribute to cancer progression, particularly in the acquisition of advanced tumorigenic and metastatic behaviour. Indeed, our previous studies demonstrated that Bit1 may function as a suppressor of metastasis *in vivo* [14]. Although the exact mechanism underlying its metastasis inhibitory effect remains to be defined, the Bit1 anoikis inducing function may play a rate limiting step in the metastasis process. Interestingly, the role of Bit1 in tumorigenesis has not been demonstrated to date.

To further address the role of Bit1 in cancer formation, we have performed preliminary online database search to examine whether Bit1 expression is altered in various types of human tumors as compared to their normal counterparts. Intriguingly, we have found that Bit1 expression is selectively and significantly suppressed in lung cancer. This preliminary finding, in conjunction with the recent evidence documenting that TLE1 may function as a lung specific oncogene [16], has prompted us to investigate the significance of the Bit1 apoptotic pathway in the anoikis insensitivity and tumorigenic potential of Non Small Cell Lung Carcinoma (NSCLC), which is the most aggressive form of lung cancer and is highly chemoresistant in part due to a deficient caspase-dependent apoptosis pathway [17], [18]. Here, we show that exogenous Bit1 circumvents the anoikis resistance of human lung adenocarcinoma A549 cells through activation of caspase-independent apoptosis and impairs their anchorage-independent growth. Consistently, knockdown of endogenous Bit1 in these cells further enhances their anoikis insensitivity and anchorage-independent potential *in vitro* and potentiates their tumorigenic growth *in vivo*. In line with its tumor suppressive function, Bit1 expression was found to be downregulated in NSCLC tumors.

## Materials and Methods

### Cell culture and transfection assays

NSCLC A549 and H460 cell lines obtained from American Type Culture Collection (ATCC) were cultured in Dulbecco's modified Eagle's medium (DMEM) with glutamine containing 10% fetal bovine serum, penicillin, and streptomycin. The stable A549 derived control and Bit1 shRNA pool of cells were maintained in Dulbecco's modified Eagle's medium (DMEM) with glutamine containing 10% fetal bovine serum, penicillin, streptomycin, and 1 µg/ml puromycin (Invitrogen). Transient transfection assays were carried out with lipofectamine 2000 (Invitrogen) for A549 cells and Lipofectamine LTX reagent (Invitrogen) for H460 cells in OPTI-MEM (Invitrogen) according to the manufacturer's protocol with the total amount of plasmid used per transfection normalized with the corresponding empty vector construct.

### Chemical reagents, antibodies, and plasmids

The Poly(2-hydroxyethyl methacrylate (Polyhema) and the mouse monoclonal anti-FLAG, anti-AES, anti-GFP and anti-B-actin antibodies were purchased from Sigma (St. Louis, MO). The polyclonal anti-TLE1 antibody was obtained from Abcam (Cambridge, MA). The caspase inhibitor zVad-fmk and the anti-myc antibody were purchased from Calbiochem (La Jolla, CA). The anti-caspase-3, anti-cleaved caspase-3, Bcl-2, Bcl-xl, Bax, Bad, and anti-PARP antibodies were obtained from Cell Signaling Technology (Beverly, MA). The mammalian expression vector encoding for mitochondrial Bit1 were generated as described previously [11]. The GFP-TLE1 plasmids were obtained from Origene (Rockville, MD). The construct encoding the cell death domain (CDD) of Bit1 was obtained from Dr. Erkki Ruoslahti (Sanford-Burnham Medical Research Institute) and was previously characterized [19].

### SiRNA and shRNA transfection

The TLE1 specific siRNAs and the control non-targeting siRNAs were purchased from Invitrogen (Carlsbad, CA). For transient transfection experiments, A549 cells (2×10^5^) were transfected with 25 µM of each siRNA using the Lipofectamine RNAiMAX transfection reagent (Invitrogen). 48 hrs post-transfection, cells were harvested and subjected to immunoblotting or anoikis assays as described below. To generate stable A549 Bit1 knockdown and control pools, A549 parental cells were transfected with pRS vector containing the short hairpin RNA against TLE1 (Origene) or the non-targeting scrambled shRNA (Origene). 48 h post-transfection, 1 µg/ml puromycin (Invitrogen) was added to the medium to select for puromycin-resistant clones. Individual puromycin-resistant clones were screened for Bit1 downregulation by immunoblotting using a specific antibody to Bit11 [15]. Two Bit1 shRNA knockdown-positive clones and two control shRNA clones were pooled for further characterization.

### Analysis of apoptosis, anoikis, and cell viability

Apoptosis was determined by quantitating the level of cytosolic nucleosomal fragments with the use of Cell Death Detection ELISA kit (Roche Molecular Biochemicals) per the manufacturer's instructions [14], [15]. To assess for anoikis cell death, cells were plated onto Polyhema coated 96 well plates in complete growth medium containing 0.5% methylcellulose at a density of 1.0×10^4^/well at various time points as previously described [14], [15]. Detached cells were then collected and subjected to the Cell Death ELISA apoptosis assay. Cell viability was quantified by alarmar blue staining (Invitrogen) and subsequent fluorescence reading (485 nm excitation wavelength and 520 nm emission wavelength) using the microplate reader (BioTek Instruments).

### Cell proliferation and soft agar assays

Anchorage-dependent growth was determined by plating cells in a volume of 150 µl at a density of 2,000 cells per well in 96-well plates. At each indicated time, the number of metabolically active cells was measured with the use of MTT assay as described previously [15]. Briefly, ten microliters of the MTT reagent at 5 mg/ml (Sigma) was added to each well in a 96-well plate. The plate was then incubated at 37°C, 5% CO2 for 3 h. Afterwards, the resulting MTT precipitate was dissolved in 100 ul of a 50% MeOH-50% DMSO solution and subjected to a 550 nm absorbance reading via a microplate reader (BioTek Instruments). The anchorage-independent growth of cells was assesed using the 96-well plate format [15]. As described previously, 5,000 cells in 0.3% agar solution was plated onto wells precoated with 0.6% agar in culture medium. The growth of the resulting colonies was quantified by alarmar blue staining (Invitrogen) and fluorescence reading at 485 nm excitation wavelength and 520 nm emission wavelength with a microplate plate reader.

### Caspase activation assay

Caspase-3 activity was determined fluorometrically using the substrate Ac-DEVD-AFC substrate and was performed according to manufacturer's instructions (Roche Applied Science).

### Protein preparation and western blotting assays

Protein preparation and western blotting were performed as described previously [14], [15]. Briefly, cells were harvested 24–48 hr after transfection with various constructs or siRNAs by adding ice-cold NP-40 lysis buffer (1% NP-40; 20 mM Tris-HCL [pH 7.4]; 150 mM NaCl; 10% glycerol, 2 mM sodium vanadate; 1 mM henylmethylsulfonyl fluoride; 10 µg/ml leupeptine; and 5 µg/ml aprotinin) and incubated at 4°C for 20 min. For immunoblot analysis, equal amounts of proteins were resolved on 4–20% gradient Tris-glycine gels (Invitrogen) and electrophoretically transferred to nitrocellulose membrane. The membranes were incubated with primary antibodies overnight at 4°C followed by secondary antibodies conjugated with horseradish peroxidase. Membranes were developed using the ECL detection system.

### Subcellular fractionation

Subcellular fractionation was performed as described previously [15]. Briefly, transfected cells cultured in attached or detached conditions at the indicated times were harvested washed once with PBS, resuspended in 1 ml of isotonic mitochondrial buffer (250 mM mannitol, 70 mM sucrose, 1 mM EDTA, 10 mM HEPES [pH 7.5]), and homogenized with 40 strokes in a Dounce homogenizer. The lysates were centrifuged at 500× g for 5 min to eliminate nuclei and unbroken cells. The supernatant was further centrifuged at 10,000× g for 30 min at 4°C to isolate the mitochondrial enriched pellet. The resulting cytosolic supernatant was subjected to SDS-PAGE electrophoresis and immunoblotting. For separation of cytosol and the nuclear fraction, the NE-PER nuclear isolation kit (Pierce, No. 78833) was used and performed as prescribed by the manufacturer [15]. The protein concentration in the different fractions was quantified using the Bio-Rad protein assay kit with BSA as the standard.

### Coimmunoprecitation assay

Coimmunoprecipitation assay was performed as described previously [15]. Briefly, transfected cells were harvested by washing once with PBS and resuspended in ice-cold Nonidet P-40 lysis buffer (1% Nonidet P-40, 20 mm Tris-HCl, pH 7.4, 150 mm NaCl, 10% glycerol, 2 mm sodium vanadate, 1 mm phenylmethylsulfonyl fluoride, 10 µg/ml leupeptin, and 5 µg/ml aprotinin) followed by a 20-min incubation at 4°C. Cell debris was removed by centrifugation. Myc-tagged Bit1 was immunoprecipitated with anti-Myc-agarose conjugate (Abcam) while GFP-tagged TLE1 was immunoprecipitated with anti-GFP-agarose (Medical and Biological Laboratories). The immunoprecipitate was thoroughly washed with lysis buffer. Bound proteins were resolved by SDS-PAGE, and Western blotting was performed using the corresponding antibody.

### *In vivo* tumorigenesis assay

All procedures were done according to protocols approved by the Institutional Committee for Use and Care of Laboratory Animals of Xavier University of Louisiana Institutional Animal Care and Use Committee (IACUC, Approval Number 060911-001BI). Eight-week-old female athymic nude mice (BALB/c) were used for the tumorigenesis assays. The A549 derived control shRNA and Bit1 shRNA cells (1.0×10^6^) were injected subcutaneously. The tumor sizes were measured periodically with a calliper, and tumor volume was determined with the formula (d1×d2^2^)/2 where d1 represents the larger diameter and d2 the smaller diameter. Mice were sacrificed when the primary tumors reached 2 cm in diameter.

### In Situ Apoptosis Detection

Detection of apoptotic cells in control shRNA and Bit1 shRNA tumor sections was performed using the DeadEnd Colorimetric TUNEL System (Promega) following the manufacturer's instructions. Briefly, sections were deparaffinized, rehydrated, and incubated with Proteinase K for 20 min at room temperature. After washing with PBS, the sections were incubated with a working concentration of recombinant Terminal Deoxynucleotidyl Transferase (rTdT) at 37°C for 1 h. The sections were the washed with PBS and immersed in 0.3% hydrogen peroxide to block endogenous peroxidase activity. The sections were subsequently washed with PBS and incubated with the streptavidin-HRP solution. The resulting dark brown signal was visualized with Diaminobenzidine (DAB) as chromogen.

### Human lung tumor tissue array analysis

Human tumor tissue array slides containing squamous cell carcinomas, adenocarcinoma, larger cell carcinoma, and matched normal lung tissues were obtained from US Biomax, Inc. (Rockville, MD). The immunohistochemistry procedure was performed by Biomax Inc. on two tissue microarray slides. As described previously [14], [15], tissue array slides were deparaffinised, hydrated and subjected to antigen retrieval. The slides were then incubated in 2.5% normal horse serum for 30 min at room temperature followed by incubation with the primary antibody (1∶100 dilution) for 1 h at room temperature. The affinity purified rabbit anti-Bit1 antibody (HPA012897) which was previously tested for its specificity [14], [15] was purchased from Sigma. Rabbit normal serum was used as negative control antibody to replace the primary antibody on control slide with 1 hr incubation. Tissue array slides were then washed and incubated with ImmPRESS reagent (Vector Laboratories) followed by treatment with peroxidise substrate DAB solution (DAKO Cytomation). The slides were scored for average Bit1 staining intensity by two investigators with no knowledge of the pathologic status of the samples. The average staining was graded as 0, no staining; 1, slight staining; 2, moderate staining; and 3, strong staining.

### Statistical analysis

Data are presented as means (±S.E.). For western blots and anoikis assays, experiments were performed at least three times with triplicates. Statistical differences between groups were established at a P value<0.05 using the two-tailed Student's t test. For lung tumor tissue array analysis, a one-way ANOVA with subsequent post hoc testing using the Tukey-Kramer multiple comparison test was used to compare the average staining intensity of each case type [14],[15]. All calculations were done using the NCSS statistical software (NCSS, Kasville, UT)

## Results

### The Anoikis Resistance of A549 cells is Associated with Lack of Significant Caspase Activity

**Figure 1:**
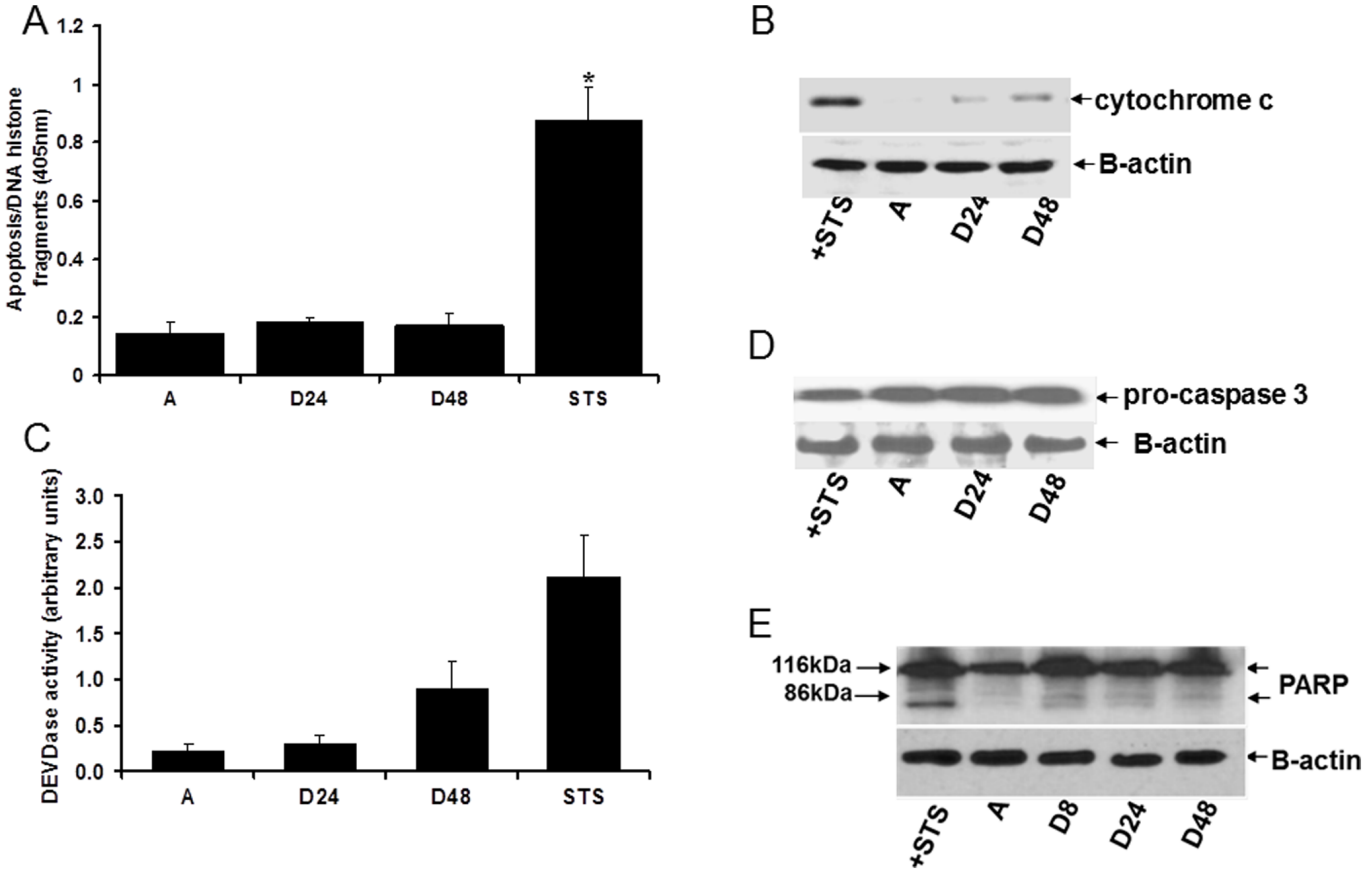
Anoikis insensitivity of A549 cells is associated with lack of caspase activation. A. Cells were cultured being attached (A) or detached from the ECM for 24 hours (D24) and 48 hours (D48) and were harvested for apoptosis analysis using the Cell Death Elisa ELISA. In parallel, cells were also treated with 1 µM staurosporine (STS) for 24 hours and subjected to Cell Death Elisa ELISA. B. Cytosolic fractions were obtained from cells in A and analysed for the presence of cytochrome c by western blot. C. The presence of caspase-3-like activity in the cell lysates from cells treated in A was determined by the cleavage of a fluorescent substrate z-DEVD-AFC (DEVD). D. and E. Cells in A were subjected to western blotting to detect the processing of pro-caspase 3 (D) and PARP (E). In A and C, three independent experiments were performed in triplicates, * indicates p<0.05 as compared to attached conditions (Student's t test).

NSCLC cells are notoriously known to be resistant to various forms of apoptotic stimuli including death receptor stimulation, cytotoxic drugs, and radiation. The ability of NSCLC to evade apoptosis has been attributed in part to an inefficient caspase-independent machinery [17], [18]. However, the mechanisms of how NSCLC blocks anoikis have not been thoroughly examined. To address the potential utility of caspase-independent mechanisms in circumventing the anoikis resistance of NSCLC cells, we first examined the contribution of the caspase pathway in anoikis. Consistent with previous reports [20], the NSCLC cell line A549 showed very low level of apoptosis when cultured in suspension (Figure 1A). Staurosporin (STS), a potent kinase inhibitor, significantly induced apoptosis in these cells (Figure 1A). To address the role of caspase dependent pathway in the anoikis blockade in A549 cells, we examined the cytosolic release of mitochondrial cytochrome c protein (a pivotal factor in activating the downstream caspase effectors) during detachment. In contrast to STS treated cells which showed substantial amounts of cytochrome c in the cytoplasm, detached cells exhibited only trace amounts of cytochrome c in the cytoplasmic fraction (Figure 1B). Next, we monitored the activation of the downstream or executioner caspase 3 via the cleavage of the fluorescent caspase-3 substrate (Figure 1C) and the processing of pro-caspase 3 through immunoblotting (Figure 1D). In line with the lack of significant cytosolic release of cytochrome c, detached A549 cells showed no substantial caspase 3 activation (Figure 1C) and displayed no processing of pro-caspase 3 (Figure 1D). The end target of the caspase machinery is to cleave the nuclear poly (ADP-ribose) polymerase (PARP) protein [5], [6]. As shown in Fig. 1E, PARP remained relatively intact in its native 116 kDa form in detached A549 cells (Figure 1E). Taken together, these findings indicate that the anoikis resistance of A549 cells is associated with an inefficient cytochrome c/caspase-dependent cell death pathway.

### Bit1 Impairs the Anoikis Resistance of Lung Carcinoma cells

**Figure 2:**
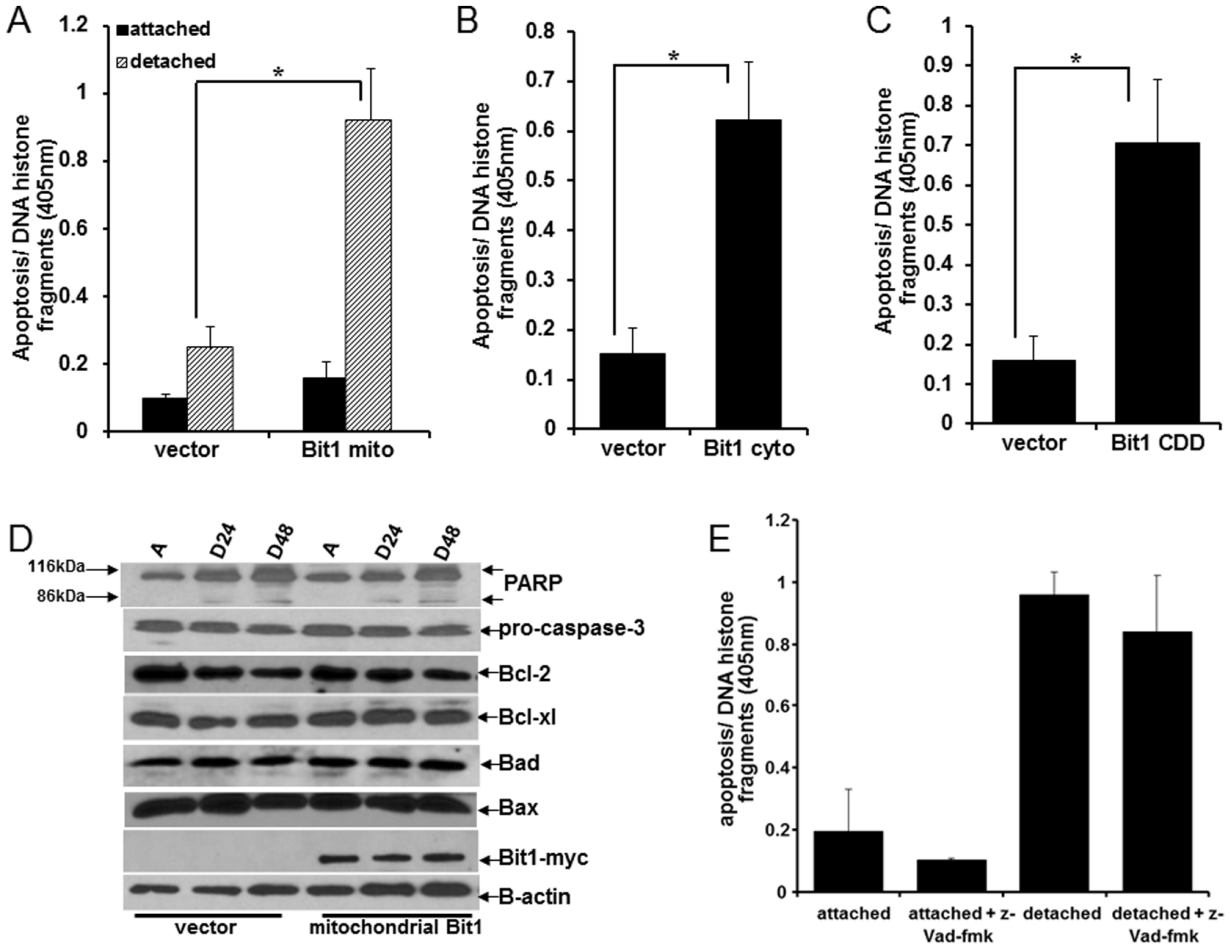
Mitochondrial Bit1 induces anoikis in A549 cells. A. Cells were transfected with the C-terminally myc-tagged mitochondrial localized Bit1 (Bit1 mito) or the empty vector construct. 24 h post-transfection, cells were cultured being attached or detached from the ECM. Following 48 h in culture, cells were harvested and analyzed for apoptosis by measuring the amount of DNA histone fragments (Cell Death Elisa). B and C. Cells were transfected with the N-terminally myc tagged cytoplasmic localized Bit1 (Bit1 cyto) (B), Bit1 cell death domain (CDD) (C), or vector construct and 48 h later, cells were subjected to Cell Death ELISA. D. Vector or Bit1 mito transfected cells were cultured being attached (A) or detached (D) from the ECM for the indicated times. Cells were then harvested and subjected to western blotting against specific antibodies to caspase-3, PARP, Bcl-2, Bcl-xl, Bax, Bad, myc, and B-actin. E. Bit1 mito transfected cells were cultured in attached or detached conditions in the presence of z-Vad-fmk (50 uM) or DMSO for 48 h. Cells were then harvested and subjected to Cell Death ELISA. In A, B, and C, three independent experiments were performed in triplicates, * indicates p<0.05 by Student's t test.

Considering the lack of significant detachment-induced caspase activation in A549 cells, we then explored the role of the Bit1 cell death pathway in the anchorage-independent survival of these cells. We increased the expression of mitochondrial Bit1 in A549 cells via transfection with a Bit1 construct that expresses the Bit1 protein exclusively in the mitochondria (Bit1 mito) [11], [15], and tested the cells' ability to survive in the absence of attachment from the ECM. The Bit1 mito and control transfected cells had the same level of spontaneous apoptosis when grown attached to a culture dish (Figure 2A). In striking contrast, the Bit1 mito transfected cells exhibited significantly increased apoptosis than the control cells upon detachment. Similar results were found when Bit1 mito was introduced into human NSCLC H460 cell line (Figure S1). To address the specificity of the anoikis induction by Bit1 mito, we targeted Bit1 expression in the cytoplasm of A549 cells with the use of a Bit1 construct that contains a tag at the N-terminal region of the Bit1 protein (Bit1 cyto) [11], [15]. Exogenous expression of cytoplasmic localized Bit1 (Bit1 cyto) in A549 (Figure 2B) and H460 (Figure S2) cells triggered apoptosis. The cell death domain (CDD) of Bit1 was recently mapped at the N-terminal 62 amino acids and was shown to be effective in inducing apoptosis in breast cancer cells [19]. Introduction of the CDD peptide also resulted in apoptosis in A549 (Figure 2C) and H460 (Figure S3) cells. Further characterization of the Bit1 anoikis pathway in A549 cells confirmed that Bit1 triggered cell death independent of caspases. First, we found no significant processing of the executioner caspase 3 in Bit1 transfected cells (Figure 2D). Second, the downstream nuclear substrate of caspases, PARP, remained intact (Figure 2D). Third, the pan-caspase inhibitor z-Vad-fmk was ineffective in blocking Bit1-induced anoikis (Figure 2E). Consistent with the lack of Bit1 effect on caspase activation, the expression of the apoptosis family of regulators that control the caspase-dependent mitochondrial apoptotic pathway was not altered by Bit1 (Figure 2D). In particular, the steady state levels of the anti-apoptotic Bcl-2 and Bcl-xl proteins as well as the pro-apoptotic Bax and Bad proteins remained unchanged by ectopic Bit1 in both attached and detached conditions. These findings indicate that Bit1 can circumvent the anoikis resistance of lung cancer cells through induction of a caspase-independent apoptotic pathway.

**Figure 3:**
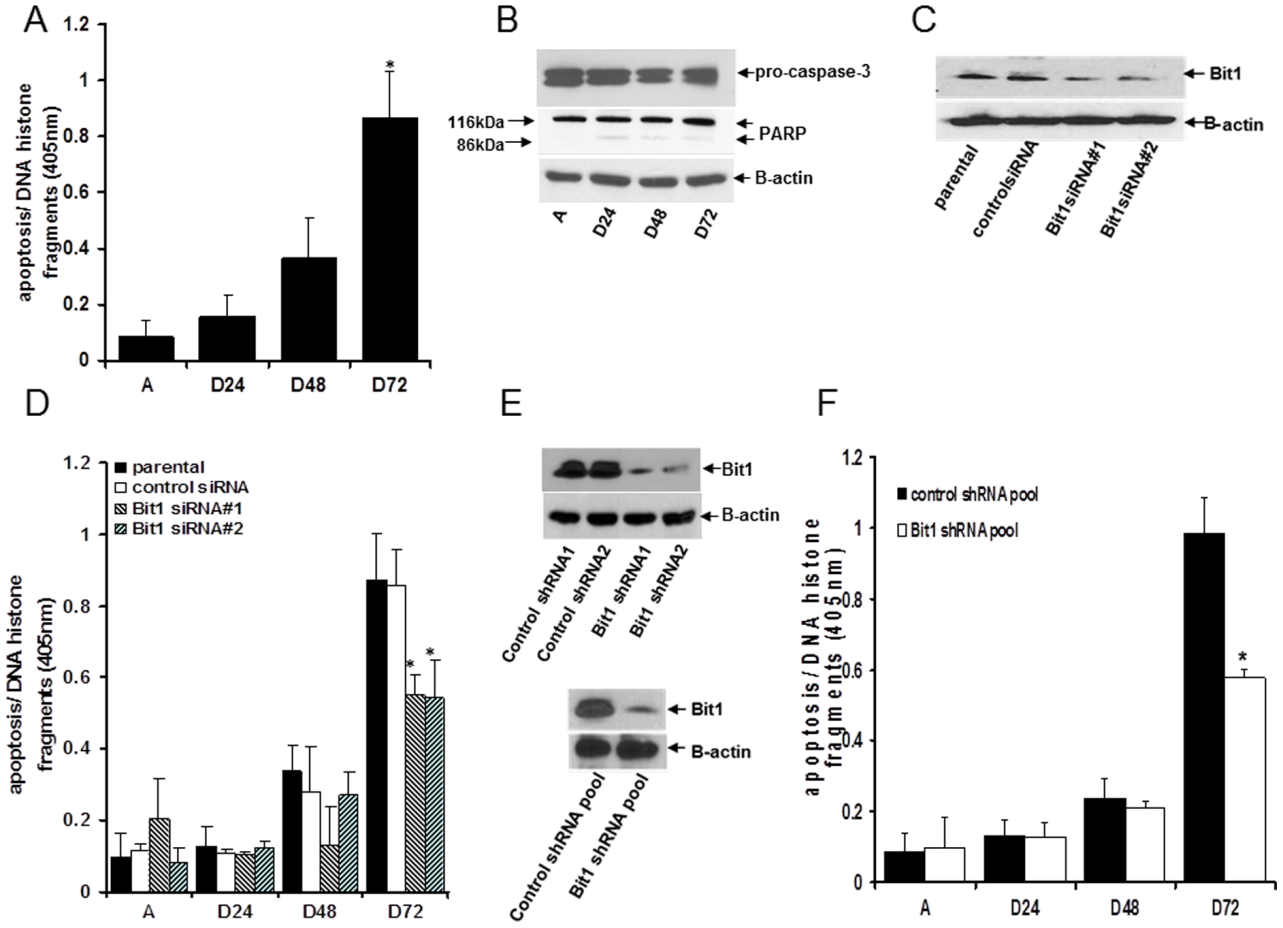
Knockdown of Bit1 expression protects A549 cells from anoikis. A. and B. Cells were cultured in attached (A) or detached (D) conditions for the indicated times and then subjected to subjected to Cell Death ELISA (A) or western blotting (B) with antibodies against caspase-3, PARP, and B-actin. In A, three independent experiments were performed in triplicates; *, p<0.05 as compared to attached cells (Student's t test). C. and D. Cells were transfected with control- or Bit1 specific siRNAs, and 48 h later, cells were subjected to immunoblotting (C) with antibodies against Bit1 and B-actin to confirm the knockdown of Bit1 expression. In parallel, control- and Bit1- siRNA treated cells were cultured in attached (A) or detached (D) conditions for the indicated times and subjected to Cell Death Elisa (D). E and F. Stable A549 control shRNA and Bit1 shRNA knockdown clones and pools were generated (as described in materials and methods) and subjected to western blotting (E) using specific antibodies to Bit1 and B-actin. In F, the control shRNA and Bit1 shRNA pools were cultured in attached (A) or detached (D) conditions for the indicated times and subsequently analysed for apoptosis by Cell Death Elisa. In A, D, and F, three independent experiments were performed in triplicates, * indicates p<0.05 as compared to control cells (Student's t test).

To further confirm the role of Bit1 as an anoikis effector in lung cancer, we examined whether knockdown of endogenous Bit1 expression will impact the anoikis insensitivity of A549 cells. We reasoned that A549 cells will undergo anoikis upon prolonged culture in suspension and if ablation of the Bit1 apoptotic pathway may provide anoikis protection. Indeed, following a protracted 72 hr culture in suspension, the detached A549 cells eventually showed evidence of apoptosis (Figure 3A). The observed anoikis induction was associated with no significant processing of caspase 3 and PARP (Figure 3B), indicating that alternative cell death mechanism(s) other than the caspase-dependent pathway is involved. We then downregulated Bit1 expression in A549 cells via short-interfering RNAs (siRNAs) and subjected the Bit1 and control siRNA treated cells to anoikis assay. Two Bit1 specific siRNAs #1 and #2 showed a significant 70–80% downregulation of Bit1 expression (Figure 3C). As compared to the A549 parental or control siRNA treated cells, the Bit1 siRNA transfected cells exhibited decreased apoptosis following a 72 h culture in suspension (Figure 3D). To complement these results, we also generated two stable Bit1 knockdown A549 derived clones, namely Bit1 shRNA1 and Bit1 shRNA2, with each clone expressing a distinct Bit1 shRNA that targets a different sequence in the 3′ noncoding region of Bit1 mRNA. In parallel, stable control shRNA clones 1 and 2 were generated from the nontargeting scrambled shRNAs. As shown in Figure 3E, the stable Bit1 shRNA1 and shRNA2 showed a reduction in Bit1 expression by 70% and 80% respectively as compared to control shRNA clones. The stable Bit1 shRNA1 and shRNA2 were then combined to generate the Bit1 shRNA pool while the control shRNA clones 1 and 2 comprised the control shRNA pool (Figure 3E). Consistent with the data derived from siRNA studies, the Bit1 shRNA pool displayed a significantly reduced level of apoptosis than the control shRNA pool following a 72 h culture in suspension (Figure 3F). Consistent with the lack of effect of ectopic Bit1 on the mitochondrial pathway (Figure 2D), the observed enhanced anoikis resistance in Bit1 knockdown cells was not associated with changes in the expression levels of the Bcl-2 family of apoptosis regulators (Figure S4). These findings, in conjunction with our results indicating that exogenous mitochondrial Bit1 can induce anoikis, provide strong evidence that the Bit1 apoptotic pathway represents an important caspase-independent anoikis mechanism in lung cancer cells.

### Bit1 is a Negative Regulator of Anchorage-independent Growth of Lung Cancer Cells

**Figure 4:**
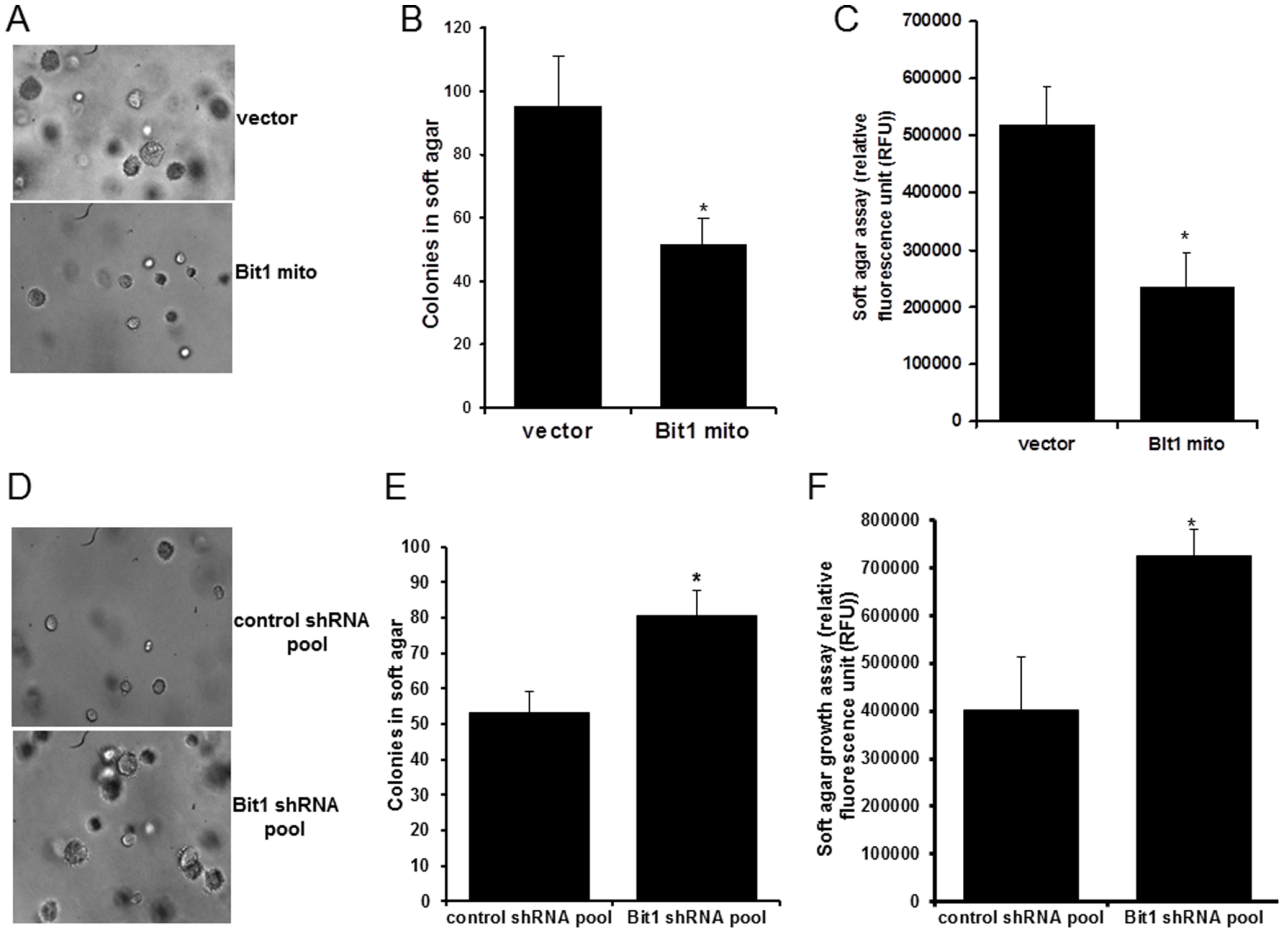
Bit1 attenuates the anchorage-independent growth of A549 cells. A., B. and C. Cells transfected with vector or Bit1 mito construct were subjected to soft agar assay as described in materials and methods. The representative colonies are shown in A. The extent of colony formation was quantified by counting the number of visible colonies with a diameter greater than 30 uM (B) and by alamar blue staining and fluorescent assay (C). D, E and F. The stable A549 control shRNA and Bit1shRNA knockdown pools were also subjected to soft agar assay. The representative colonies are visualized in A and quantified by counting the number of visible colonies (E) and alamar blue assay (F). In B, C, E and F, three independent experiments were performed in triplicates, * indicates p<0.05 as compared to corresponding control cells (Student's t test).

The anchorage-independent growth potential of malignant cells is dependent on their ability to resist anoikis [1], [2]. Here, we investigated whether Bit1 can regulate the anchorage-independent growth capability of A549 cells using an *in vitro* soft agar assay. As shown in Figures 4A and 4B, the vector transfected cells exhibited numerous colonies in soft agar after two week incubation. In stark contrast, mitochondrial Bit1 treated cells showed a reduction in the number and sizes of colonies, suggesting impairment of their anchorage-independent growth. The compromised growth of Bit1 transfected cells on semisolid medium was further quantified by alamar blue staining (Figure 4C).

We also tested the ability of Bit1 shRNA and control shRNA pools to grow in an anchorage-independent environment. Consistent with their enhanced anoikis resistance, the stable Bit1 shRNA pool showed greater ability to grow in soft agar than the control shRNA pool (Figures 4D and 4E). Following a shorter period of 10 day incubation, it was evident that the Bit1 shRNA pool exhibited a greater number of larger colonies as compared to control shRNA pool. The enhanced anchorage-independent growth ability of Bit1 shRNA pool was quantified by alamar blue assay (Figure 4F). It is noteworthy that in monolayer culture the Bit1 shRNA and control shRNA pools exhibited similar anchorage-dependent growth kinetics (Figure S5). Collectively, these results indicate a role of Bit1 in suppressing the anchorage-independent growth potential of lung cancer A549 cells.

### TLE1 Blocks Bit1-induced Anoikis by Sequestering AES in the Nucleus

**Figure 5:**
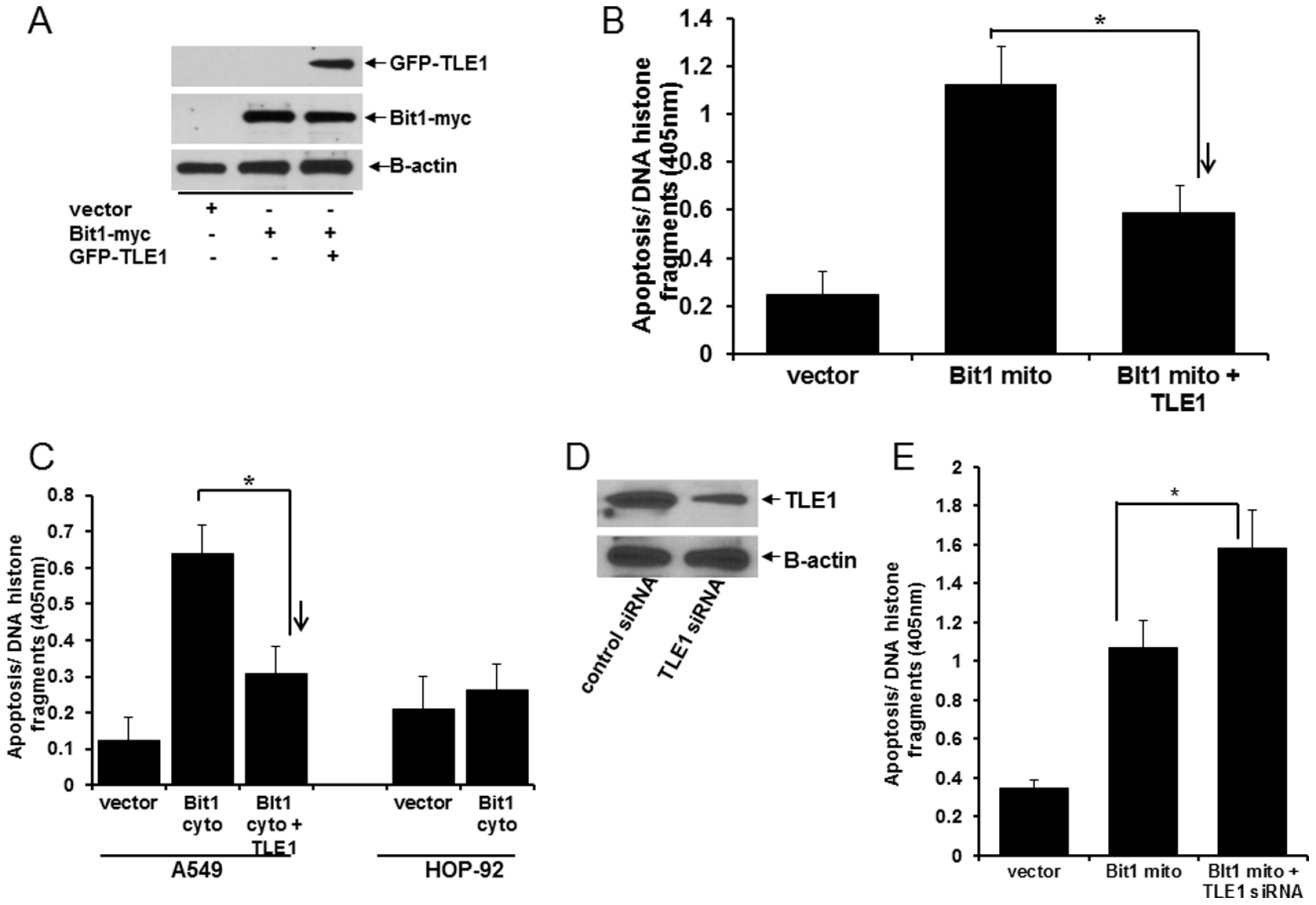
TLE1 inhibits Bit1 anoikis function. A. and B. A549 cells were transfected with Bit1 mito and/or with GFP-tagged TLE1 construct. 24 h post transfection, the expression of exogenous Bit1 and TLE1 in transfected cells was confirmed by western blotting using specific antibodies to myc and GFP tags. The amount of plasmid transfected into cells was normalized with the vector construct. In parallel, transfected cells were grown in suspension for 48 h and then subjected to Cell Death ELISA assay (B). C. A549 can HOP-92 cells were transfected with Bit1 cyto or GFP-TLE1 construct. 48 h post-transfection, cells were harvested and analyzed for apoptosis by Cell Death Elisa. D. A549 cells were transfected with control- or TLE1 specific siRNAs, and 48 h later, cells were subjected to immunoblotting with antibodies against TLE1 and B-actin to confirm the knockdown of TLE1 expression. E. A549 cells were transfected with vector or Bit1 mito construct, and 24 hr later, the mitochondrial Bit1 treated cells were transfected with control- or TLE1-siRNAs as indicated. Cells were then cultured in suspension for 48 hr and subjected to Cell Death Elisa. In B, C, and E, three independent experiments were performed in triplicates, * indicates p<0.05 by Student's t test.

In addition to integrin mediated cell attachment, the only other anti-apoptotic factor that can block Bit1 apoptosis is the groucho corepressor TLE1 protein [11]. TLE1 has recently been shown to be a lung specific oncogene [16], but its underlying oncogenic function has not been established. Here, we examined whether TLE1 may protect lung cancer cells from anoikis induced by Bit1. To address this possibility, A549 cells were cotransfected with C-terminally myc-tagged Bit1 and/or full length GFP-tagged TLE1 construct and then cultured on poly-HEMA coated plates. The ectopic expression of mitochondrial Bit1 and TLE1 was confirmed by immunoblotting (Figure 5A). Consistent with our previous findings ([11]–[15], Figure 2A), detachment induced a higher level of apoptosis in Bit1 transfected cells than in control cells (Figure 5B). Importantly, exogenous TLE1 attenuated the high level of anoikis in mitochondrial Bit1 transfected cells. Furthermore, ectopic TLE1 also inhibited the apoptosis induced by targeted expression of Bit1 in the cytoplasm (Bit1 cyto) in A549 cells (Figure 5C). To further substantiate the protective effect of TLE1 on Bit1 anoikis, we downregulated endogenous TLE1 expression and examined its effect on Bit1 anoikis function. Knockdown endogenous TLE1 expression in A549 cells was performed via a specific TLE1 siRNA SMART pool. The TLE1 siRNA duplexes showed a significant 50–60% downregulation of endogenous TLE1 expression in A549 cells (Figure 5D). The efficacy and specificity of the TLE1 siRNAs in downregulating TLE1 protein was also confirmed in cells expressing a GFP-tagged human TLE1 (Figure S6). Suppression of endogenous TLE1 resulted in increased susceptibility of A549 cells to Bit1-induced anoikis (Figure 5E). Collectively, these findings indicate a critical role of TLE1 in suppressing the Bit1 anoikis pathway in A549 cells.

**Figure 6:**
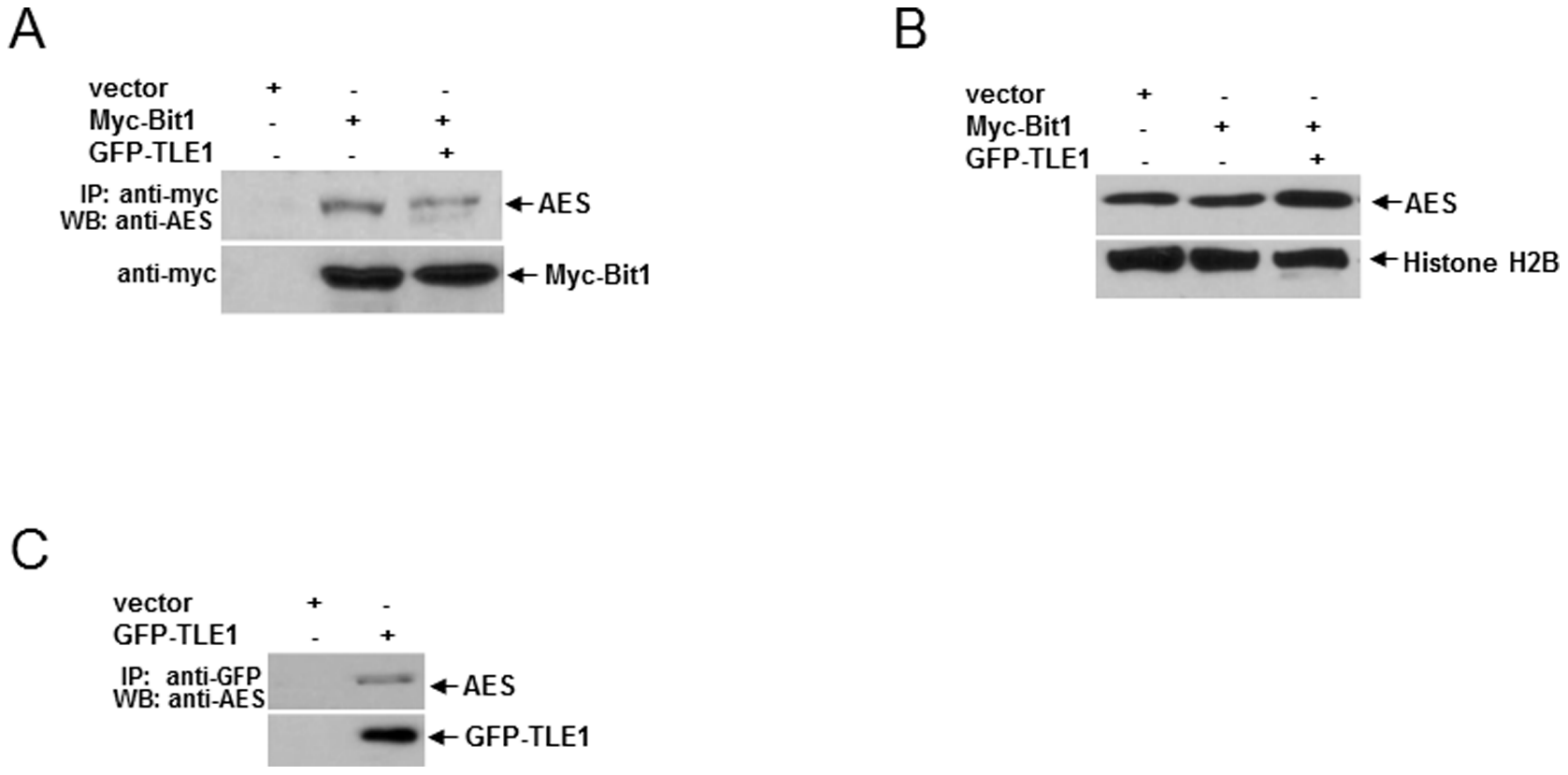
TLE1 blocks Bit1-AES complex formation by sequestering AES in the nucleus. A. and B. A549 cells were transfected with GFP-TLE1 together with N-terminally myc tagged Bit1 construct. The amount of DNA was normalized with the empty vector in each transfection. 24 h post-transfection, cells were harvested and cell extracts were prepared, immunoprecipitated with agarose-immobilized anti-myc, and immunoblotted with anti-AES and anti-myc antibodies (A). In parallel, transfected cells were subjected to nuclear isolation as indicated in the materials and methods. The resulting nuclear fraction was run on SDS-PAGE and western blotted with an anti-AES antibody. The Histone H2B was used as a nuclear marker (B). C. A549 cells were transfected with vector or GFP-TLE1 construct, and 24 h post-transfection, cells were subjected to immunoprecipitation with agarose-immobilized anti-GFP and western blotting with anti-AES and anti-GFP antibodies.

Following its release to the cytoplasm, mitochondrial Bit1 binds to AES and the resulting Bit1-AES complex is the pro-apoptotic component that mediates the Bit1 apoptotic effect [11]. Consistent with this notion, the AES null lung cancer cell line HOP-92 remained resistant to Bit1-mediated apoptosis (Figure 5C). To address the mechanism underlying the inhibitory effect of TLE1 on Bit1 anoikis function, we examined whether nuclear TLE1 may inhibit the Bit1-AES complex formation by competitive binding to and sequestering AES protein (which normally shuttles between the nuclear and cytoplasmic compartments [21], [22]) in the nucleus. Exogenous TLE1 expression decreased the levels of Bit1-AES complex (Figure 6A) and resulted in significant enrichment of AES in the nucleus (Figure 6B). The sequestration of AES in the nucleus by TLE1 was in part mediated by the association of TLE1 with AES (Figure 6C) as previously documented [21], [22]. Taken together, these findings indicate TLE1 is a suppressor of the Bit1 anoikis pathway through nuclear sequestration of AES.

### Downregulation of Bit1 Promotes Tumorigenicity in Nude Mice

**Figure 7:**
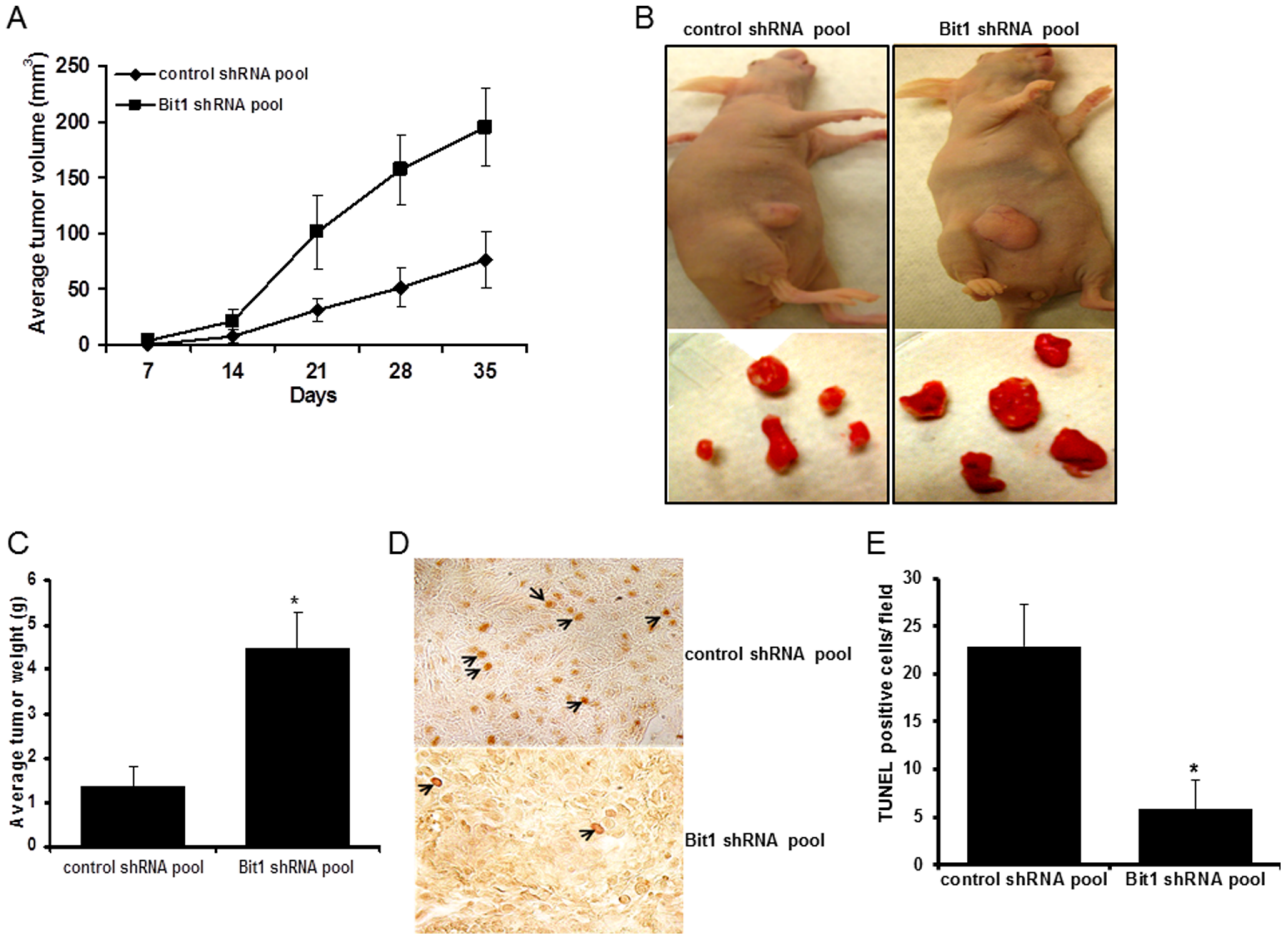
Suppression of Bit1 enhances tumorigenecity of A549 cells *in vivo*. A. A549 derived control shRNA and Bit1 shRNA cells (1.0×10^6^) were injected subcutaneously in 8 week old BALB/c nude mice. Tumors were measured periodically with a calliper on the days after injection as indicated. B. and C. Mice were anesthetized and sacrificed on the day 35 after injection. Subcutaneous tumors were surgically excised, and the tumors were then photographed (the representative tumors are shown in (B) and weighed (C). In D and E, tissue sections of control shRNA and Bit1 shRNA xenografts were subjected to TUNEL assay to detect cellular apoptosis. Representative images of TUNEL stained tumor sections are shown in (C). Arrows indicate apoptotic cells characterized by a brown staining. D. TUNEL positive tumor cells in serial sections of control shRNA and Bit1 shRNA tumors were quantified. In C and E, * indicates p<0.05 as compared to control tumors (Student's t test).

To address the role of Bit1 on tumor growth *in vivo*, we subcutaneously injected control shRNA and Bit1 shRNA clonal pools in nude mice. As shown in Figure 7A, we observed a significant increase in the growth rate of Bit1 shRNA tumors as compared to control shRNA tumors as assessed by two independent experiments. Seven weeks post-injection, the tumors were surgically excised and weighed (Figures 7B and 7C). The average weight of Bit1 shRNA tumors was approximately 3-fold heavier than the control shRNA tumors (Figure 7C). These findings indicate that loss of Bit1 expression confers enhanced tumorigenicity of A549 cells in a mouse xenograft model.

As malignant cells form a subcutaneous three-dimensional tumor *in vivo*, a significant proportion of these cells are deprived of proper contacts with the basement membrane and subject to apoptosis. Based on the ability of the Bit1 cell death pathway to effectively trigger apoptosis and anoikis in lung cancer cells *in vitro* (Figures 2A–2C) and the recent report demonstrating that exogenous Bit1 CDD induces apoptosis in human breast tumor xenografts *in vivo* [19], the level of apoptosis in control and Bit1 knockdown tumor xenografts was evaluated by subjecting serial tumor tissue sections to in situ TUNEL assay (Figures 7D and 7E). The TUNEL-stained control tumor sections exhibited localized apoptosis as evidenced by the presence of numerous apoptotic cells particularly in the middle of the tumors. In contrast, fewer apoptotic cells were observed in Bit1 knockdown tumors (Figures 7D and 7E). These findings suggest that the enhancement of tumorigenicity in Bit1 knockdown cells is in part due to reduced basal apoptosis and are in agreement with previous published data showing strong inhibition of breast tumor xenograft growth *in vivo* by ectopic Bit1 CDD treatment [19].

### Bit1 is Downregulated in Human Lung Tumors

**Figure 8:**
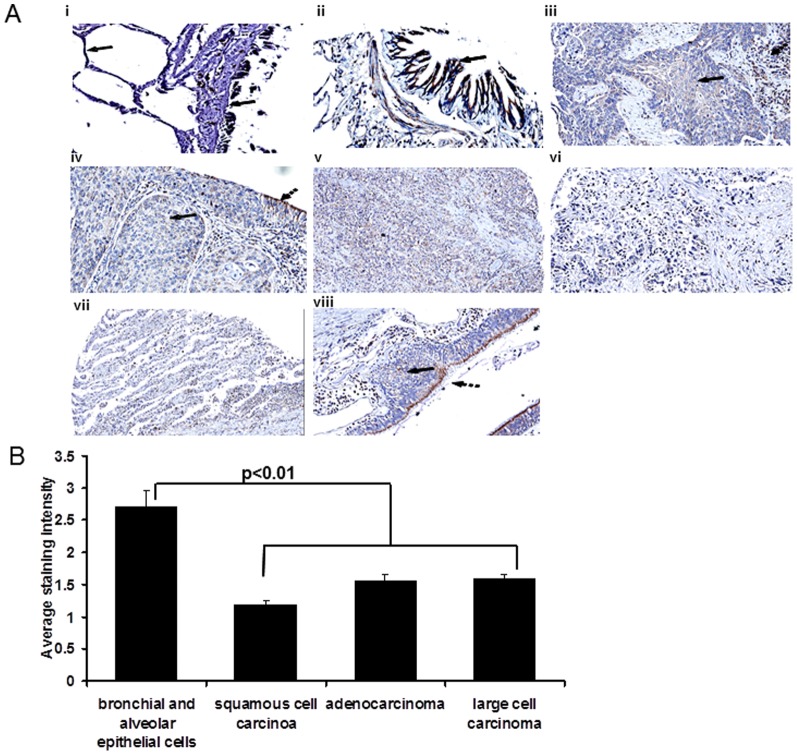
Bit1 is downregulated in NSCLC tissues. A. Lung tumor tissue array slides were stained with the affinity purified rabbit anti-Bit1 antibody. Images are representative of each respective case type: normal lung (i, ii 10×), squamous cell carcinoma (iii, iv, viii 10×), adenocarcinoma (v, vi 10×) and large cell carcinoma (vii). B. The average Bit1 immunostaining intensity in normal bronchial and alveolar epithelial tissues and in various types of NSCLC cancer was determined as described in materials and methods. The normal bronchial and alveolar epithelial tissues was statistically significant from the NSCLC tumors using the ANOVA and subsequent Tukey post-hoc analysis (*, P<0.05).

Based on our findings that downregulation of Bit1 expression enhances the *in vivo* tumorigenecity of A549 cells, we examined the possibility that Bit1 expression is suppressed in human lung cancers. Immunohistochemistry staining of human lung tumor tissue microarrays was performed using an affinity purified polyclonal antihuman Bit1 antibody [14], [15]. In normal lung, the columnar epithelial cells lining the bronchial mocusa and the alveolar epithelial cells showed strong cytoplasmic immunoreactivity for Bit1 (Figure 8Ai,ii, indicated by the bolded arrow). Using the strong immunoreactivity of the normal bronchial and alveolar epithelial cells as an internal control, cytoplasmic staining of the tumor cells from multiple cores was evaluated. Immunoreactivity was graded as 0 (none), 1 slight, 2 (moderate), or 3 (strong) as described in materials and methods. Significant suppression of Bit1 (grade 0 and 1) was found in 14 of 20 (70%) of the squamous cell carcinomas (Figure 8Aiii,iv, viii), 40 of 71 (56%) of the adenocarcinomas (Figure 8Av,vi), and in 4 of 8 (50%) of the large cell undifferentiated carcinomas (Figure 8Avii). Interestingly, we found that in some of the NSCLC lesions residual Bit1 expression is present or retained in epithelial cells closest to the lumen (Figure 8A iv and viii, unbolded arrow) as well as in nearby inflammatory cells (Figure 8Aiii, unbolded arrow) while Bit1 expression is completely lost in more dysplastic cells that have invaded the underlying connective tissue (Figure 8A iii, iv and viii, bolded arrow). Figure 8B shows the average Bit1 immunostaining intensity in various types of NSCLC tissues as compared to normal bronchial and alveolar epithelial cells. These findings indicate that Bit1 expression is selectively lost in a fraction of various types of NSCLCs and suggest that loss of Bit1 may contribute to lung tumorigenesis.

## Discussion

Bit1 is a mitochondrial protein which is part of an apoptosis pathway that is uniquely regulated by integrin-mediated cell attachment [11]. Following loss of cell attachment, Bit1 is released to the cytosol to induce a caspase-independent form of apoptosis, which can only be suppressed by integrin-mediated cell adhesion. Considering that cell attachment appears to be the only up-stream treatment that can effectively block Bit1 apoptosis, Bit1 may play a special role in anoikis as documented in several cellular model systems [11]–[15]. Since anoikis resistance is a critical determinant of transformation, we hypothesize that suppression of Bit1 function is an alternative mechanism by which malignant cells resist anoikis and endow themselves with enhanced anchorage-independent growth potential *in vitro* and tumorigenecity *in vivo*. We were particularly interested in examining the role of Bit1 in lung tumorigenesis based on existing evidence indicating that the Bit1 apoptotic pathway is nonfunctional in lung cancer. Through the NIH genomics and ONCOMINE database, we found that the Bit1 mRNA expression (via electronic northern and SAGE analysis) is significantly downregulated in human lung cancer relative to its normal counterpart. The loss of Bit1 expression in human lung tumors was subsequently confirmed via immunohistochemistry staining of lung tumor tissue arrays (Figure 8). A recent study indicated that TLE1, the sole known inhibitor of Bit1 apoptotic function, is a putative lung specific oncogene and is overexpressed in human lung tumors [16]. Downregulation of Bit1 expression and/or overexpression of TLE1 are potential mechanisms that could lead to disruption of the Bit1 pathway. In this report, we provide the first evidence of the tumor suppressive function of Bit1. Our findings also indicate that a potential mechanism underlying the TLE1 oncogenic function is to protect lung cancer cells from Bit1 induced anoikis.

A critical determinant of tumor aggressiveness and progression is the acquisition of anoikis resistance by malignant cells. Two molecular pathways are involved in the induction of anoikis, and both converge on caspase activation to effect nuclear signs of apoptosis [5]–[9]. Considering the pivotal role of caspases in anoikis induction, defect or inactivation of caspases could be a major contributory factor in the emergence of anoikis insensitivity of cancer cells. Indeed, we show here that the anoikis resistance of A549 NSCLC cells is associated with lack of significant mitochondrial release of cytochrome c and caspase activity. The latter was evidenced by the absence of processing and cleavage of the executioner caspase 3 and the downstream nuclear PARP protein (Figure 1). These findings are consistent with previous reports demonstrating that the chemoresistance of NSCLC is in part due to a deficiency in cytochrome c/caspase-dependent apoptotic pathway [17], [18]. Despite the compromised caspase-dependent pathway, we show here that exogenous mitochondrial Bit1 can resensitize NSCLC cells to anoikis. Furthermore, downregulating endogenous Bit1 expression in NSCLC cells further enhances their anoikis insensitivity after a prolonged culture in suspension. Hence, the loss of Bit1 expression in human lung tumors (Figure 8) may represent an important mechanism by which NSCLC cells circumvent anoikis.

The molecular mechanism underlying Bit1 anoikis function remains to be fully elucidated. Following its release to the cytoplasm as a result of loss of cell attachment, Bit1 forms a complex with the transcriptional regulator protein Amino-terminal Enhancer of Split (AES) [11]. The formation and abundance of the Bit1/AES complex is likely critical to Bit1 apoptotic activity [11]–[15]. We have shown previously that the level of the Bit1-AES complex determines the extent of Bit1-mediated apoptosis and is regulated by the cytoplasmic concentration of Bit1 and AES. While AES exists in both the cytoplasmic and nuclear compartments [21], [22], the presence and/or concentration of Bit1 in the cytoplasm will likely serve as a rate determining event in the Bit1/AES complex formation. Cytoplasmic Bit1 presence may result from incomplete import of Bit1 into mitochondria (such as through forced expression of Bit1 tagged at the N-terminus (Bit1 cyto)) or its release from mitochondria as a result of compromised mitochondrial membrane stability (such as in cells undergoing anoikis). As documented in our previous studies [11]–[15], the formation of the pro-apoptotic Bit1-AES complex is negatively regulated by the nuclear TLE1 corepressor at least in part by sequestering AES in the nucleus (Figure 6). To further examine how the Bit1/AES complex induces apoptosis, our current research work focuses on the potential regulation of the TLE1 transcriptional program by the Bit1/AES complex. Numerous studies have reported that TLE1 exhibits pro-survival and antiapoptotic function in several cellular models [23]–[25]. Hence, it is conceivable that the nuclear TLE1 corepressor controls an anti-apoptotic transcriptional program. Shutting off the TLE1 anti-apoptotic transcriptional machinery may represent an important molecular event underlying Bit1 apoptosis. It is interesting to speculate that suppression of the TLE1 transcriptional machinery by the Bit1/AES axis may lead to expression and/or activation of nuclear enzymes that could effect DNA fragmentation and nuclear signs of apoptosis. Thus, characterization of the anti-apoptotic TLE1 transcriptional program via identification of the downstream target genes may provide mechanistic insights into the Bit1 apoptotic pathway.

The TLE1 corepressor has recently been shown to be a putative lung oncogene [16]. Allen et al. 2006 found that transgenic mice carrying the human TLE1 homologue, Grg1, develop lung adenocarcinomas. Consistent with its lung oncogenic function, TLE1 was found to be upregulated in a significant fraction of human lung carcinomas. The molecular mechanism and cellular basis of TLE1 oncogenic function, however, remain to be determined. In this report, we show that TLE1 functions to protect lung cancer cells from Bit1-induced anoikis. While downregulation of endogenous TLE1 expression is sufficient to enhance the sensitivity of NSCLC cells to Bit1 anoikis, exogenous TLE1 attenuated Bit1 anoikis. The ability of TLE1 to protect NSCLC cells from Bit1 anoikis may provide impetus for induction and enhancement of anchorage-independent growth. Considering that anchorage-independent potential is a determinant of transformation and tumorigenesis, the ability of TLE1 to block the Bit1 anoikis pathway may contribute to its oncogenic function in lung carcinoma.

Avoidance of apoptosis is one of the hallmarks of cancer, and the molecular pathways of this process are not fully understood. In particular, the role of alternative caspase-independent cell death mechanisms in tumorigenesis has not been documented. Here, we show evidence that the integrin-regulated caspase-independent apoptosis effector Bit1 exerts a tumor suppressive effect in lung carcinoma. Downregulation of Bit1 expression in the human NSCLC A549 cell line is sufficient to enhance colony formation in soft agar and accelerate tumor growth in immunocompromised animals. Consistent with its tumor suppressor function, Bit1 expression is downregulated in advanced stages of lung carcinoma. It is interesting to note that disruption of the Bit1 pathway can also be achieved via overexpression of its inhibitor TLE1, which has been shown to be upregulated in human lung cancer [16]. As a potential tumor suppressor of lung cancer, selective targeting of Bit1 in lung tumors may serve as a therapeutic strategy to reactivate the apoptotic mechanism in lung cancer cells and block tumor progression. In line with this notion, selective tumor delivery of the cell death domain (CDD) of Bit1 was shown to be effective in regressing the growth of caspase-resistant breast tumors in xenograft models [19]. Currently, we are examining the utility of the CDD peptide in inhibiting the growth of lung carcinomas *in vivo*. Given the importance of anoikis resistance in tumor progression and metastasis, understanding how the Bit1 apoptotic pathway may impact lung cancer metastasis remains an important area of investigation. Indeed, we have previously shown Bit1 may function as an inhibitor of metastasis in melanoma and breast tumors [14]. The significant loss of Bit1 expression in the highly aggressive human NSCLC tissues as shown in this report underscores the potential *in vivo* role of Bit1 in lung metastatic disease.

In conclusion, we have found that the Bit1 apoptotic pathway is a viable cell death pathway that can circumvent the anoikis resistance of NSCLC. While targeting Bit1 expression in the mitochondria inhibits anoikis resistance and anchorage-independent growth of NSCLC cells, specific downregulation of endogenous Bit1 in these cells further potentiated their anoikis insensitivity and anchorage-independent growth *in vitro* and tumorigenic growth *in vivo*. The findings of this study provide the first evidence in support of Bit1 as a potential tumor suppressor in human lung cancer and raise the possibility that the previously reported oncogenic function of TLE1 is to attenuate the Bit1 anoikis pathway.

## Supporting Information

Figure S1 **Exogenous mitochondrial Bit1 (Bit1 mito) expression induces anoikis in H460 cells.** Cells were transfected with the C-terminally myc-tagged mitochondrial localized Bit1 (Bit1 mito) or the empty vector construct. 24 h post-transfection, cells were cultured being attached or detached from the ECM. Following 48 h in culture, cells were harvested and analyzed for apoptosis by measuring the amount of DNA histone fragments (Cell Death Elisa). Three independent experiments were performed in triplicates, * indicates p<0.05 by Student's t test.
(TIF)

Figure S2 **Exogenous expression of cytoplasmic localized Bit1 induces apoptosis in H460 cells.** Cells were transfected with the N-terminally myc tagged cytoplasmic localized Bit1 (Bit1 cyto) or vector construct and 48 h later, cells were subjected to Cell Death ELISA. Three independent experiments were performed in triplicates, * indicates p<0.05 as compared to control cells (Student's t test).
(TIF)

Figure S3 **Ectopic expression of Bit1 CDD peptide induces apoptosis in H460 cells.** Cells were transfected with the N-terminally myc tagged Bit1 cell death domain (CDD) or vector construct and 48 h later, cells were subjected to Cell Death ELISA. Three independent experiments were performed in triplicates, * indicates p<0.05 as compared to control cells (Student's t test).
(TIF)

Figure S4 **Knockdown of Bit1 does not alter the expression levels of the Bcl-2 family of proteins in A549 cells.** Stable A549 derived control shRNA and Bit1 shRNA pool of cells were cultured in attached (A) or detached (D) conditions for the indicated time and subsequently subjected to western blotting with antibodies against Bcl-2, Bcl-xl, Bax, Bad, Bit1, and B-actin.
(TIF)

Figure S5 **Knockdown of Bit1 does not alter the anchorage-dependent growth of A549 cells.** Stable A549 derived control shRNA and Bit1 shRNA pool of cells were plated onto regular tissue culture plates and the growth of cells was quantified by MTT assay at the indicated time points.
(TIF)

Figure S6 **Exogenous GFP-TLE1 is downregulated by the specific TLE1siRNAs.** A549 cells were transfected with the vector or GFP-TLE1 construct, and 24 h later cells were transfected with control- or TLE1 specific siRNAs as indicated. 36 h post-siRNA transfection, cells were subjected to immunoblotting with antibodies against GFP and B-actin to confirm the knockdown of exogenous TLE1 expression.
(TIF)
