# Supplementary figures and images for: The Anoikis Effector Bit1 Displays Tumor Suppressive Function in Lung Cancer Cells
Source: PLoS One. 2014 Jul 8;9(7):e101564. doi: 10.1371/journal.pone.0101564 (PMC4086906; doi:10.1371/journal.pone.0101564)

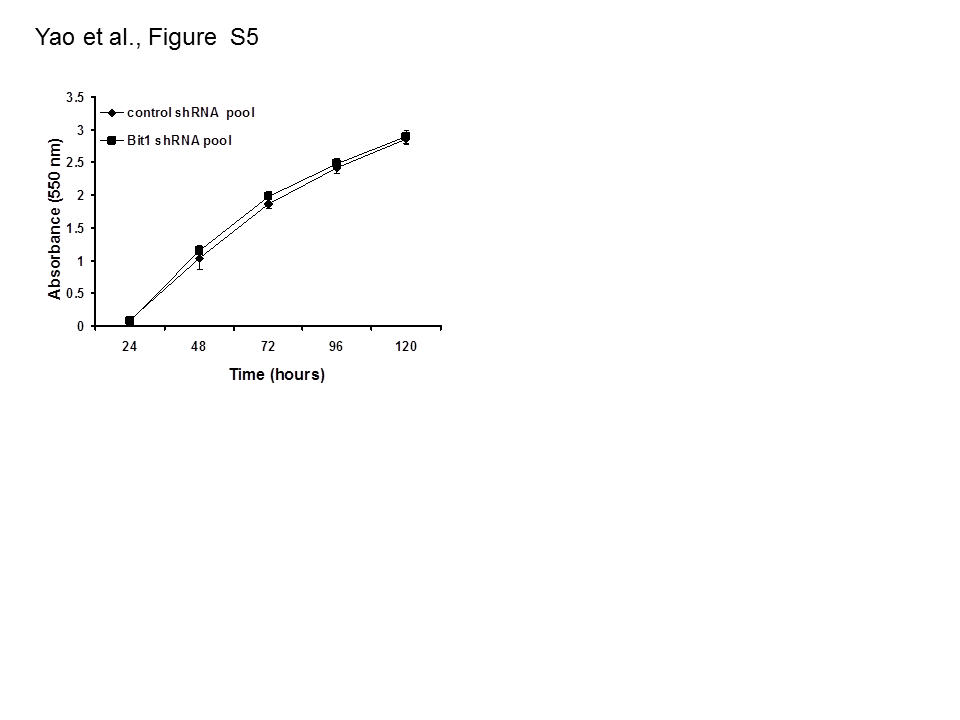

Supplement: Figure S5 — Knockdown of Bit1 does not alter the anchorage-dependent growth of A549 cells. Stable A549 derived control shRNA and Bit1 shRNA pool of cells were plated onto regular tissue culture plates and the growth of cells was quantified by MTT assay at the indicated time points. (TIF) [file pone.0101564.s005.tif]
